# Diagnostic performance of rapid screening tests for asymptomatic bacteriuria among pregnant women in a tertiary care hospital in India

**DOI:** 10.1038/s41598-026-50193-y

**Published:** 2026-04-28

**Authors:** Sheetal Gouda, Sooraj Mohan, Shobhit Kumar Dwivedi, P. Dinesha, V. N. Venkatesh, Sushant S. Satputaley

**Affiliations:** 1 Department of Microbiology, Karwar Institute of Medical Sciences, Karwar, 581301 India; 2 Manipal Institute of Technology, Manipal Academy of Higher Education, Manipal, 576104 India; 3 Department of Mechanical Engineering, St. Vincent Pallotti College of Engineering & Technology, Nagpur, 441108 India

**Keywords:** Maternal health, Antenatal screening, Infectious diseases, Health equity, Preventive care, Biomarkers, Diseases, Health care, Medical research, Microbiology

## Abstract

Asymptomatic bacteriuria (ASB) during pregnancy is associated with adverse maternal and foetal outcomes, necessitating early and reliable detection. While urine culture remains the reference standard, its limited availability and delayed turnaround time highlight the need for effective rapid screening tests. This study aimed to evaluate the clinicodemographic profile, microbiological spectrum, and diagnostic performance of selected rapid screening tests for early detection of ASB among pregnant women attending a tertiary care centre in India. A cross-sectional study was conducted among 97 antenatal women. Clinicodemographic and comorbidity data were collected using a structured questionnaire and analysed for association with culture-confirmed asymptomatic bacteriuria. Midstream urine samples were subjected to wet mount microscopy, Gram staining, catalase testing, and semi-quantitative urine culture. Culture positivity was defined as ≥ 105 CFU/mL of a single uropathogen. Diagnostic indices, including sensitivity, specificity, positive predictive value (PPV), and negative predictive value (NPV), were calculated for individual tests and their parallel combinations. The prevalence of ASB was 22.7%. Staphylococcus aureus was the predominant isolate. Wet mount microscopy and catalase testing demonstrated 100% sensitivity and NPV, whereas Gram staining showed high specificity (97.3%) and PPV (90.0%). Parallel combinations of screening tests did not improve diagnostic performance beyond individual high-performing tests. Simple, rapid screening tests, particularly wet mount microscopy and catalase testing, are effective for ruling out ASB in pregnancy, while Gram staining serves as a reliable confirmatory test. Rational selection of screening tools can enhance antenatal ASB detection, especially in resource-limited settings.

**Supplementary Information:**

The online version contains supplementary material available at 10.1038/s41598-026-50193-y.

## Introduction

Ensuring maternal health through early detection and prevention of infectious diseases is central to Sustainable Development Goal 3(United Nations^1^, which emphasizes good health and well-being for women and children. Urinary tract infection (UTI) is more common yet poses a potential risk in pregnant women due to physiological and immunological changes (Ansaldi & Martinez de Tejada Weber, 2023). Asymptomatic bacteriuria (ASB) is a clinical entity where an individual has no symptoms of UTI, but urine culture reveals a colony count of > 10^5^ CFU/mL of urine of a single type of pathogenic bacteria^2^. Pregnant women with bacteriuria have a higher incidence of developing UTI and pyelonephritis, in turn exhibiting adverse effects on the pregnancy and birth outcomes^3^. The prevalence of ASB among antenatal cases in India ranged from 8 to 45%^4–6^. Diabetes mellitus^7^, renal abnormalities^8^, bladder dysfunctions^9^, low socio-economic status (SES)^10^, and medical interventions like catheterization^11^ are associated with a higher risk of developing ASB. However, the association of ASB with maternal age, type of delivery, gestational age, and gravida number is still not clear^12^.

Rapid and early diagnosis of ASB has reported a reduction in prenatal and postnatal complications like premature rupture of membranes, preterm labour, low-birth-weight babies, postpartum UTI, and postpartum endometritis^13–16^. The urine culture remains the gold standard test for diagnosing ASB. However, important pathogens like Chlamydiae and Mycoplasma are often missed in culture, and the turnaround time is around 18–24 h for culture and an additional 18 h for antibiotic susceptibility testing (AST)^17–19^. Many screening tests, like wet mount^20^, Gram stain^21^, catalase, leukocyte esterase^22^, nitrate-reduction^22^, are a few of the prominent ones that are available for rapid detection of ASB.

The sensitivity, specificity, positive predictive value (PPV), and negative predictive value (NPV) are considered as output variables in the literature. The comparative analysis of various screening tests suggested the efficacy of Gram staining or catalase test as comparable with that of urine culture; however, the data on the combination of these tests are unavailable^23,24^. There is no single test that can diagnose ASB rapidly and accurately. The prediction and rapid diagnosis of ASB involves both clinical and laboratory analysis using some robust technology, which can predict bacteriuria. Despite the availability of multiple rapid screening tests for ASB, considerable heterogeneity remains in the reported diagnostic performance across studies, particularly with respect to sensitivity, specificity, and predictive values in pregnant populations (Ansaldi & Martinez de Tejada Weber, 2023;P. G. Patel et al.^25^,. Most existing investigations have evaluated individual screening tests in isolation, with limited emphasis on their comparative performance within the same cohort. Moreover, evidence on the diagnostic utility of combining screening tests in a parallel manner to enhance early detection while minimizing missed cases is sparse. Data integrating clinicodemographic risk factors with microbiological profiles and diagnostic test performance are also limited, especially from tertiary care settings in coastal regions of India. This lack of consolidated evidence hampers the development of pragmatic, resource-efficient screening algorithms for routine antenatal care.

In this context, the present study was undertaken to evaluate the clinicodemographic characteristics associated with ASB among pregnant women attending a tertiary care centre in Karwar, India, and determine the microbiological profile of uropathogens causing ASB. The study also assesses the diagnostic performance of commonly used rapid screening tests, including wet mount microscopy, Gram stain, and catalase test, using urine culture as the reference standard, and analyzes the diagnostic utility of selected combinations of these screening tests for the early and reliable detection of ASB in pregnancy.

## Methods

### Study design and setting

A cross-sectional study was conducted at the Department of Obstetrics and Gynaecology and the Department of Microbiology for a period of 6 months. Ethical approval was obtained from the Institutional Ethics Committee of Karwar Institute of Medical Sciences, Karwar, India (Ref. no. IEC/KRIMS/O/80/2025-26), and the study was conducted in accordance with relevant guidelines and regulations.

### Sample collection

The sample size was calculated based on the expected prevalence of asymptomatic bacteriuria among pregnant women. A prevalence of 8.47% ($\:p$) was considered, as reported in a previous Indian study^4^, with a 95% confidence level ($\:Z$ = 1.96) and an absolute precision of 6% ($\:d$) commonly used in pregnancy-related, single-centre studies^4,26^. The minimum required sample size was estimated using the formula, $\:n=\frac{{Z}^{2}p(1-p)}{{d}^{2}}$. Accordingly, the calculated sample size was 83. To improve the robustness of subgroup analyses and to account for potential exclusions due to inadequate or contaminated urine samples, the sample size was increased by approximately 15%, resulting in a final study population of 97 pregnant women. This sample size was considered adequate for estimating prevalence and evaluating the diagnostic performance of screening tests with acceptable precision. Informed and written consent was obtained from all the pregnant women participating in the study. The data on clinical and demographic information were obtained using a structured questionnaire. The urine samples of the patients were collected; however, the patients were not involved in the study design, selection of outcome measures, data analysis, interpretation of results, or dissemination of findings.

All pregnant women with no symptoms of urinary tract infection were included; among them, those who had renal abnormalities, bladder dysfunction, or were on antibiotic therapy were excluded from the study. The relevant history was obtained via a questionnaire to analyse any association between clinicodemographic profile and ASB. Further, they were instructed to collect clean catch, mid-stream urine in a wide-mouthed universal container following aseptic precautions. The samples thus obtained were transported within 2 h to the Microbiology department for performing culture and screening tests. The urine culture and colony count interpretation were performed independently by microbiology personnel who were blinded to the results of the screening tests and to participants’ clinical information. Similarly, the personnel performing and interpreting the screening tests were blinded to urine culture results.

### Culture test

The urine was inoculated into blood agar and MacConkey agar using a 4 mm diameter loop and incubated at 37 °C overnight. Any plates showing a predominant single type colony count > 10^5^ CFU/mL were considered an ASB positive case. The positive samples were further identified based on colony morphology, Gram staining, and standard biochemical tests, including catalase, coagulase, oxidase, and selected sugar fermentation tests, as appropriate for Gram-positive and Gram-negative organisms, following standard microbiological procedures. Antibiotic susceptibility testing was performed using the Kirby-Bauer disk diffusion method on Mueller–Hinton agar, and results were interpreted according to Clinical and Laboratory Standards Institute guidelines. However, antibiotic susceptibility results were not included in the present analysis and are therefore not reported in this study.

### Gram stain

A loop of uncentrifuged, well-mixed urine was placed on a glass slide and air-dried. Gram stain was performed following standard protocol^27^, and the smear was seen microscopically under oil immersion field (OIF). Even a single bacterium in 20 OIF was considered significant as it correlates to a colony count of 10^5^ CFU/mL. The findings were recorded in a spreadsheet.

### Wet mount preparation

Around 0.05 ml of well-mixed, uncentrifuged urine sample was placed on a slide, a coverslip was added, and observed in low power followed by high power field (HPF). ≥5 pus cells/HPF was considered significant. The number of pus cells per sample was recorded in a spreadsheet.

### Catalase test

Around 2 ml of urine was taken in a test tube. 4 drops of 10% hydrogen peroxide were added with gentle shaking for 5 s. The test was declared positive if the entire ring or layer of effervescence was observed within a minute on the surface of urine in the test tube.

### Clinicodemographic and comorbidity data collection

Clinicodemographic data, including age, gestational age, parity, socioeconomic status, and relevant comorbidities (anaemia, diabetes mellitus, hypertension, prior UTI, and catheterization), were collected using a structured questionnaire. Comorbid conditions were also recorded based on the structured questionnaire. The questionnaire used for data collection is provided as Supplementary File 1.

### Statistical analysis

Statistical analysis of clinicodemographic data was performed using Python and Microsoft Excel software to determine any association between these factors and ASB. The data obtained from screening tests were compared with culture results to determine sensitivity, specificity, positive predictive value, and negative predictive value. Pearson’s chi-square test was used to evaluate the association between each categorical predictor and culture positivity. For each predictor, a contingency table was constructed with predictor categories as rows and culture status (positive/negative) as columns. The chi-square statistic (χ²), degrees of freedom (df), and two-tailed p-value were calculated.

### Instruments, reagents, and software

Microscopic examinations were performed using a standard laboratory light microscope (Olympus, Japan). Culture media, including blood agar and MacConkey agar, and biochemical reagents were procured from HiMedia Laboratories Pvt. Ltd., India. Gram-staining reagents and hydrogen peroxide for catalase testing were obtained from standard commercial suppliers. Data analysis was carried out using Python and Microsoft Excel.

## Results and discussion

### Epidemiology

A total of 97 antenatal urine samples were analysed for ASB, using the laboratory criterion that only growth representing ≥ 10^5^ CFU/mL is considered a culture-positive case of ASB, and any colony counts below this threshold are considered negative. 22 samples (22.68%) were culture-positive in this study. The maternal age and gestational age ranged from 20 years to 36 years and 16 to 39 weeks, with a mean maternal and gestational age of 28 years and 30.9 weeks, respectively. The highest prevalence of ASB was noted in the 3rd trimester (90.9%), followed by the 2nd trimester (9.1%), while no reports were recorded in the 1 st trimester. Similar trimester-wise trends have been reported in previous studies, where the third trimester is associated with increased urinary stasis and hormonal influences, predisposing to higher bacteriuria rates^28^. The relatively lower prevalence observed in the 2nd trimester may be attributed to transitional physiological changes during pregnancy, where urinary stasis and hormonal influences begin to increase but have not yet reached the peak levels typically seen in the third trimester, thereby resulting in comparatively lower bacterial colonization^29–31^.

The prevalence of ASB in pregnant women in the community is reported in the range of 2–10%, but is comparable to hospital-based studies from similar settings in India and other developing countries, where higher rates have been attributed to referral bias and high-risk pregnancy profiles^28,32,33^. The higher prevalence (22.67%) in this tertiary care hospital-based study could be due to population characteristics and study-related factors. Tertiary care hospitals often deal with high-risk pregnancies, including women with anaemia, previous UTIs, diabetes, hypertensive disorders, lower socioeconomic status, and poor nutritional status. Such conditions predispose to lower immunity, urinary stasis, and increased bacterial colonization, thereby increasing the prevalence of ASB compared to community based study. In addition, pregnant women attending tertiary care hospitals are more likely to have prior antibiotic exposure, repeated urine testing, or hospitalization, which may alter the urinary flora and increase detection rates, which might have resulted in an elevated prevalence rate in the present study.

### Influence of socioeconomic status on ASB

Among 22 positive cases, 81.8% had low socioeconomic status (SES), defined based on indicators such as income level, occupation, and living conditions as recorded in the structured questionnaire. SES is a proxy for determinants that affect hygiene, nutrition, antenatal care access, living conditions, and exposure to community pathogens. Low SES can correlate with crowded living conditions, limited access to clean water, and delayed antenatal visits; all of which increase the risk of colonization and untreated asymptomatic infection. Examining SES allows assessment of social determinants in the local context. However, the χ² test statistic obtained a value of 1.27 with a high p-value of 0.736, revealing no significant association in this cohort. Similar reports have also been found in the literature^34–36^. However, other studies have reported a significant association between low socioeconomic status and ASB^37,38^, highlighting the role of hygiene and access to antenatal care.

### Influence of the presence of comorbidities on ASB

Among 22 positive cases, anaemia (9.1%), prior urinary tract infection (9.1%), and catheterization (9.1%) were the most common comorbidities, followed by diabetes mellitus (4.5%), while no cases of hypertensive disorder (0%) were observed. Comorbid conditions (anaemia, diabetes, hypertension, and prior UTIs) have direct biological plausibility linking them to ASB. Diabetes and glycosuria create an environment favourable to bacterial proliferation^39,40^. Anaemia and chronic medical conditions may impair immunologic defenses^41,42^. History of previous UTI indicates prior colonization and may indicate persistent uropathogen reservoirs^43,44^.

The results revealed a χ² statistic of 13.50, with a p value of 0.00024 (df = 1), indicating a statistically significant association between any comorbidity and culture positivity. 9/22 (40.9%) of positives had comorbid conditions as shown in Table 1. The effect size and low p-value support the clinical plausibility that coexisting medical conditions increase ASB risk, consistent with mechanistic insights such as impaired host defence (anaemia), glycosuria (diabetes), and prior urinary tract disruption (previous UTIs). The distribution of positives across SES categories suggests that in our specific population, clinical susceptibility (comorbidity) is a stronger driver of ASB than SES.

**Table 1:** Chi-square contingency table to determine the association between comorbidities and Culture positivity.

Comorbidity present?	Culture Negative	Culture Positive	Total
No	70	5	75
Yes	13	9	22
**Total**	**83**	**22**	**97**

### Influence of catheter use on ASB

Urinary catheterization is an established risk factor for bacteriuria through direct introduction of organisms and biofilm formation^45^. Although catheter use was rare in our cohort, it is clinically relevant and therefore was tested for association. A high probability value (0.37) with a χ² value of 1.99 revealed an insignificant association. Due to data limitations, it is inferred that catheterization was infrequent, limiting statistical power.

### Influence of parity on ASB

Parity was analyzed because obstetric history may be associated with pelvic floor changes, prior instrumentation, and differential prenatal care access^46^; primigravidae sometimes have different healthcare behaviour and vulnerability compared to multigravidae. It is a customary demographic variable in obstetric infection studies. Similar to SES and catheter usage, parity also did not reveal any significant association for ASB, with χ² = 1.03, and a p-value of 0.31.

### Microbiological profile of the samples

**Fig. 1:**
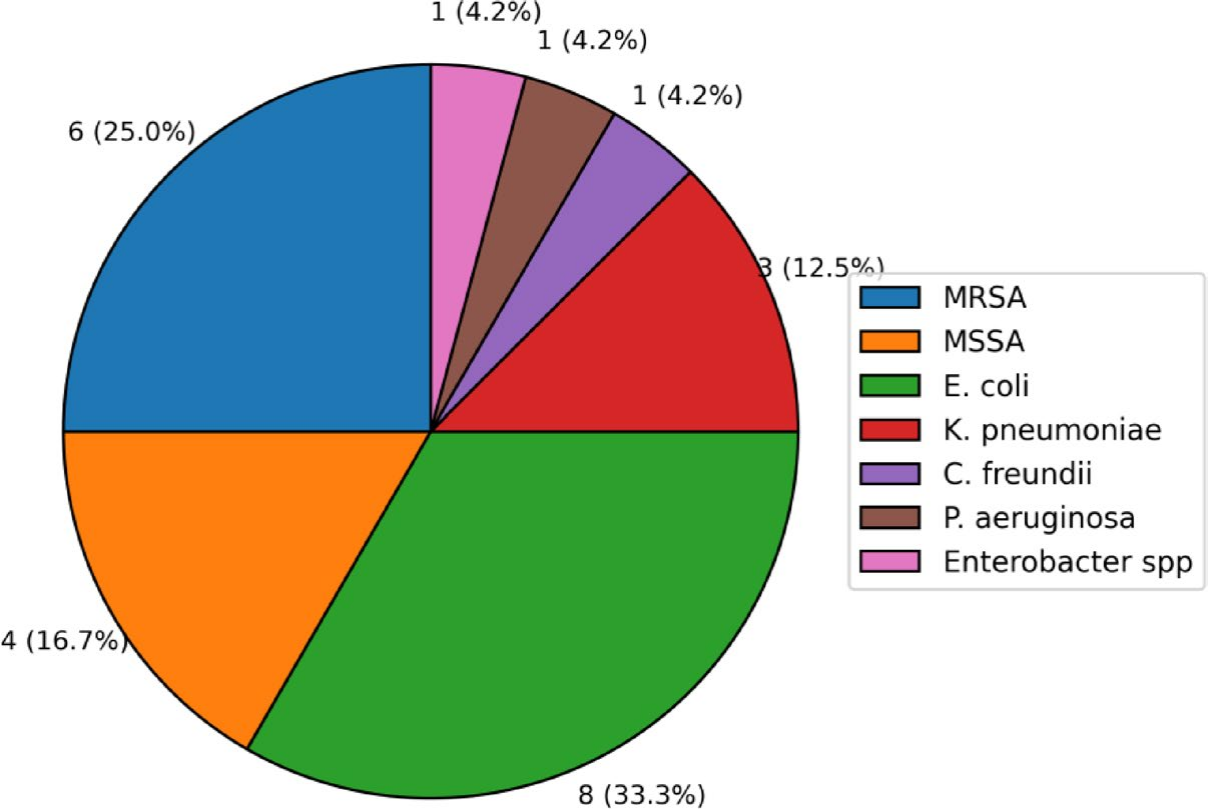
Distribution of the different organism frequency among the culture-positive samples.

*Staphylococcus aureus* (41.7%) was the predominant organism isolated, followed by *Escherichia coli* (33.3%) and *Klebsiella pneumoniae* (12.5%). Other isolates included *Citrobacter freundii*,* Pseudomonas aeruginosa*, and *Enterobacter spp.*, each accounting for 4.2% of isolates (Fig. 1). Staphylococci are the normal commensals of the skin and perineal region, and hence can easily colonize the lower urinary tract without producing overt disease^47^. They have relatively low virulence and lack efficient uropathogenic factors compared to *E. coli*. As a result, their presence in urine often leads to colonization rather than tissue invasion or inflammation. In addition, Staphylococci are potent biofilm producers, especially in catheterized individuals, allowing them to persist in the urinary tract while evading the host immune response^48^. In contrast, *E. coli* expresses toxins and an inflammatory response, which triggers the immune system, leading to symptoms like dysuria, burning micturition, and urgency, making it more commonly associated with symptomatic UTI compared to ASB^49^. The presence of *Citrobacter freundii*^50^, *Pseudomonas aeruginosa*^51^, and *Enterobacter spp.*^52^, although less frequent, highlights a heterogeneous microbial spectrum; though not typical community uropathogens, they may reflect prior antibiotic pressure or healthcare exposures.

### Diagnostic tests for the detection of ASB

The comparative diagnostic evaluation of wet mount microscopy, Gram staining, and catalase testing, both individually and in parallel combinations, reveals distinct performance profiles that have important implications for ASB screening strategies. The consolidated diagnostic indices for all tests and combinations are summarized in Table 2.

Wet mount microscopy demonstrated perfect sensitivity (100.0%) and NPV (100.0%), along with moderate specificity (82.7%) and PPV (62.9%), indicating that all culture-positive ASB cases were correctly identified and that a negative wet mount reliably excluded infection. This finding supports the utility of pyuria as a sensitive surrogate marker of urinary tract inflammation. However, the moderate specificity and PPV indicate that pyuria is not specific to bacteriuria meeting the diagnostic culture threshold. Inflammatory conditions unrelated to bacterial infection, low-grade colonization, or specimen contamination may contribute to false-positive findings. Consequently, while wet mount microscopy is highly effective as a screening tool to rule out ASB, its standalone diagnostic accuracy for confirming infection remains limited. Similar high sensitivity of pyuria-based screening has been reported in previous studies, supporting its role as an effective rule-out test, although specificity remains limited^53,54^.

**Table 2:** Diagnostic performance of individual tests and parallel combinations for detection of asymptomatic bacteriuria with culture as the reference standard.

Test	Sensitivity (%)	Specificity (%)	PPV (%)	NPV (%)
Wet mount microscopy	100.0	82.7	62.9	100.0
Gram stain	81.8	97.3	90.0	94.8
Catalase test	100.0	80.0	59.5	100.0
Gram stain + Catalase	100.0	80.0	59.5	100.0
Gram stain + Wet mount	100.0	82.7	62.9	100.0
Catalase + Wet mount	100.0	80.0	59.5	100.0
Gram + Catalase + Wet mount	100.0	80.0	59.5	100.0

Gram staining exhibited a contrasting diagnostic pattern characterized by high specificity (97.3%) and PPV (90.0%), with comparatively lower sensitivity (81.8%) and NPV (94.8%). A positive Gram stain strongly predicted culture positivity, reflecting the presence of a sufficient bacterial burden to allow microscopic visualization. This reinforces the value of Gram staining as a confirmatory or rule-in test. However, the reduced sensitivity indicates that a subset of culture-positive cases may escape detection, likely due to bacterial concentrations below the visual threshold or uneven organism distribution in uncentrifuged urine. These characteristics limit the suitability of Gram staining as a primary screening test, particularly in asymptomatic populations where bacterial loads may be marginal. These findings are consistent with prior studies that have demonstrated high specificity of Gram staining, making it a useful confirmatory tool despite lower sensitivity^55^.

Catalase testing demonstrated diagnostic behaviour closely resembling wet mount microscopy, with perfect sensitivity (100.0%) and NPV (100.0%), but lower specificity (80.0%) and PPV (59.5%). The absence of false-negative results confirms its effectiveness in excluding ASB when negative. The reduced specificity likely reflects catalase activity originating not only from urinary pathogens but also from inflammatory cells and contaminating organisms. Despite this limitation, the rapid turnaround time, minimal technical requirements, and excellent rule-out capability position catalase testing as a practical screening modality, especially in high-volume or resource-limited clinical settings. Previous studies have also reported the utility of catalase testing as a rapid screening tool, although its specificity is limited due to non-bacterial sources of catalase activity^56^.

Parallel testing strategies were evaluated to determine whether combining tests could overcome individual limitations. When Gram stain was combined with catalase testing using an “OR” rule, diagnostic indices remained identical to those of catalase alone. Sensitivity and NPV stayed at 100%, while specificity and PPV showed no improvement. This indicates that all culture-positive cases detected by Gram staining were already captured by catalase testing, resulting in complete overlap and no incremental diagnostic gain. The addition of Gram staining in this context therefore, increases procedural complexity without improving case detection.

A similar pattern was observed with the combination of Gram stain and wet mount microscopy. Although sensitivity and NPV remained perfect, specificity and PPV were unchanged compared with wet mount alone. This suggests that Gram stain-positive cases were a subset of wet mount-positive cases in this cohort. As a result, the parallel use of these two tests did not enhance diagnostic discrimination and failed to reduce the false-positive burden inherent to pyuria-based screening.

The combination of catalase testing and wet mount microscopy likewise failed to improve diagnostic performance beyond catalase alone. While perfect sensitivity and NPV were preserved, specificity and PPV remained moderate. This outcome reflects the dominant contribution of catalase testing in identifying all culture-positive cases, with wet mount microscopy providing no additional discriminatory value under a parallel testing strategy.

Extending the parallel approach to include all three tests, Gram stain, catalase, and wet mount microscopy, produced identical diagnostic indices to catalase alone. Sensitivity and NPV remained at 100%, while specificity and PPV showed no improvement. This finding confirms that expanding the test panel does not necessarily translate into superior diagnostic performance when the component tests detect largely overlapping subsets of true-positive cases. Instead, such combinations may amplify false-positive results, leading to unnecessary confirmatory cultures and increased clinical workload.

Collectively, the diagnostic indices summarized in Table 2 illustrate a clear functional hierarchy among the evaluated tests. Wet mount microscopy and catalase testing function optimally as exclusionary tools due to their perfect sensitivity and NPV, whereas Gram staining provides strong confirmatory value owing to its high specificity and PPV. The absence of incremental benefit from parallel combinations highlights the importance of rational test selection rather than indiscriminate test aggregation. These findings support a selective, stepwise diagnostic framework in which highly sensitive tests are employed for initial screening and highly specific tests are reserved for confirmation.

### Limitations of the study

The study was conducted at a single centre, with a limited sample size. Small cell counts for some contingency categories limit power for certain associations. A combined comorbidity variable increases power but obscures the relative contributions of specific conditions (diabetes vs. anaemia vs. hypertension). There is an inherent lack of antibiotic susceptibility reporting in the current dataset. Such data are critical for clinical recommendations and will be required for treatment guidance.

### Strengths and limitations of this study

- Culture-based reference standard ensured methodological validity of diagnostic comparisons.
- Uniform testing on identical samples allowed reliable head-to-head evaluation of screening methods.
- Assessment of parallel test combinations addressed a key methodological gap in ASB screening studies.
- Single-centre, cross-sectional design limits external generalizability and temporal inference.
- Restricted laboratory test spectrum excludes enzymatic strips and molecular diagnostics.

## Conclusions

Rapid screening tests play an important role in the early detection of asymptomatic bacteriuria in pregnancy. Wet mount microscopy and catalase testing demonstrated high sensitivity and negative predictive value, making them effective screening tools, while Gram staining showed high specificity and positive predictive value, supporting its role as a confirmatory test. Parallel combinations of tests did not provide additional diagnostic benefit. Rational selection of simple screening methods can improve ASB detection, particularly in resource-limited settings.

## Supplementary Information

Below is the link to the electronic supplementary material.

Supplementary Material 1

## Data Availability

The data may be made available on reasonable request.

## Declarations

### Transparency

The lead author affirms that the manuscript is an honest, accurate, and transparent account of the study being reported; that no important aspects of the study have been omitted; and that any discrepancies from the study as planned have been explained.

### Patient and public involvement

Informed and written consent was obtained from all the pregnant women participating in the study. The data on clinical and demographic information were obtained using a structured questionnaire. The urine samples of the patients were collected; however, the patients were not involved in the study design, selection of outcome measures, data analysis, interpretation of results, or dissemination of findings.

## References

[1] United Nations. *The Sustainable Development Goals Report 2025*. (2025).

[2] Tadesse, S. et al. Prevalence, antimicrobial susceptibility profile and predictors of asymptomatic bacteriuria among pregnant women in Adigrat General Hospital, Northern Ethiopia. *BMC Res. Notes* **11**(1), 740. 10.1186/s13104-018-3844-1 (2018). 30340646 10.1186/s13104-018-3844-1 PMC6194591

[3] de Souza, H. D., Diório, G. R. M., Peres, S. V., Francisco, R. P. V. & Galletta, M. A. K. Bacterial profile and prevalence of urinary tract infections in pregnant women in Latin America: a systematic review and meta-analysis. *BMC Pregnancy Childbirth*. **23** (1), 774. 10.1186/s12884-023-06060-z (2023). 37940852 10.1186/s12884-023-06060-z PMC10631168

[4] Awasthi, A., Adiga, P. & Rao, S. Prevalence of asymptomatic bacteriuria and sterile pyuria in pregnant women attending antenatal clinic in a tertiary care center in Karnataka: A pilot study. *Clin. Epidemiol. Glob. Health.* **1**(1), 44–49. 10.1016/j.cegh.2012.11.001 (2013).

[5] Khan, S., Rashmi, Singh, P., Siddiqui, Z. & Ansari, M. Pregnancy-associated asymptomatic bacteriuria and drug resistance. *J. Taibah Univ. Med. Sci.* **10** (3), 340–345. 10.1016/j.jtumed.2015.01.011 (2015).

[6] Menezes, E. V. et al. Reducing stillbirths: prevention and management of medical disorders and infections during pregnancy. *BMC Pregnancy Childbirth*. **9** (S1), S4. 10.1186/1471-2393-9-S1-S4 (2009). 19426467 10.1186/1471-2393-9-S1-S4 PMC2679410

[7] Laway, B. A., Nabi, T., Bhat, M. H. & Fomda, B. A. Prevalence, clinical profile and follow up of asymptomatic bacteriuria in patients with type 2 diabetes-prospective case control study in Srinagar, India. *Diabetes Metab. Syndr. Clin. Res. Rev.* **15**(1), 455–459. 10.1016/j.dsx.2020.12.043 (2021). 10.1016/j.dsx.2020.12.043 33601179

[8] Ross, J. & Hickling, D. Medical treatment for urinary tract infections. *Urol. Clin. North Am.* **49**(2), 283–297. 10.1016/j.ucl.2021.12.004 (2022). 35428434 10.1016/j.ucl.2021.12.004

[9] Nicolle, L. E. Urinary tract infections in the older adult. *Clin. Geriatr. Med.* **32**(3), 523–538. 10.1016/j.cger.2016.03.002 (2016). 27394021 10.1016/j.cger.2016.03.002

[10] Adegoke, A. A., Ikott, W. E. & Okoh, A. I. Carbapenem resistance associated with coliuria among outpatient and hospitalised urology patients. *New Microbes New Infect.* **48**, 101019. 10.1016/j.nmni.2022.101019 (2022). 36176538 10.1016/j.nmni.2022.101019 PMC9513764

[11] Kulbay, A., Joelsson-Alm, E., Amilon, K. & Tammelin, A. Asymptomatic bacteriuria and urinary tract infection in geriatric inpatients after indwelling urinary catheter removal: a descriptive two-centre study. *Infect. Prev. Pract.* **6** (4), 100411. 10.1016/j.infpip.2024.100411 (2024). 39583881 10.1016/j.infpip.2024.100411 PMC11582739

[12] Ansaldi, Y., Martinez, T. & Weber, B. Urinary tract infections in pregnancy. *Clin. Microbiol. Infect.* **29** (10), 1249–1253. 10.1016/j.cmi.2022.08.015 (2023). 36031053 10.1016/j.cmi.2022.08.015

[13] Karikari, A. B., Saba, C. K. S. & Yamik, D. Y. Assessment of asymptomatic bacteriuria and sterile pyuria among antenatal attendants in hospitals in northern Ghana. *BMC Pregnancy Childbirth*. **20** (1), 239. 10.1186/s12884-020-02936-6 (2020). 32321461 10.1186/s12884-020-02936-6 PMC7178963

[14] Kazemier, B. M. et al. Maternal and neonatal consequences of treated and untreated asymptomatic bacteriuria in pregnancy: a prospective cohort study with an embedded randomised controlled trial. *Lancet. Infect. Dis*. **15** (11), 1324–1333. 10.1016/S1473-3099(15)00070-5 (2015). 26255208 10.1016/S1473-3099(15)00070-5

[15] Kodikara, H., Seneviratne, H., Kaluarachchi, A. & Corea, E. Diagnostic accuracy of nitrite dipstick testing for the detection of bacteriuria of pregnancy. *Public. Health*. **123** (5), 393–394. 10.1016/j.puhe.2009.01.007 (2009). 19394058 10.1016/j.puhe.2009.01.007

[16] Wang, A., Nizran, P., Malone, M. A. & Riley, T. Urinary tract infections. *Prim. Care Clin. Office Pract.* **40**(3), 687–706. 10.1016/j.pop.2013.06.005 (2013). 10.1016/j.pop.2013.06.005 23958364

[17] Hisada, K. et al. Development and evaluation of a novel quenching probe PCR (GENECUBE) assay for rapidly detecting and distinguishing between Chlamydia pneumoniae and Chlamydia psittaci. *J. Microbiol. Methods*. **184**, 106212. 10.1016/j.mimet.2021.106212 (2021). 33781806 10.1016/j.mimet.2021.106212

[18] Pence, M. A., McElvania TeKippe, E. & Burnham, C.-A. Diagnostic assays for identification of microorganisms and antimicrobial resistance determinants directly from positive blood culture broth. *Clin. Lab. Med.* **33**(3), 651–684. 10.1016/j.cll.2013.03.010 (2013). 23931843 10.1016/j.cll.2013.03.010

[19] Redelinghuys, M. J., Ehlers, M. M., Dreyer, A. W., Lombaard, H. A. & Kock, M. M. Comparison of the new Mycofast Revolution assay with a molecular assay for the detection of genital mycoplasmas from clinical specimens. *BMC Infect. Dis.* **13**(1), 453. 10.1186/1471-2334-13-453 (2013). 24079603 10.1186/1471-2334-13-453 PMC3849776

[20] Szmulik, M., Trześniewska-Ofiara, Z., Mendrycka, M. & Woźniak-Kosek, A. A novel approach to screening and managing the urinary tract infections suspected sample in the general human population. *Front. Cell. Infect. Microbiol.*10.3389/fcimb.2022.915288 (2022). 36093203 10.3389/fcimb.2022.915288 PMC9455924

[21] Suzuki, N. & Isobe, N. Determining causal pathogens and inflammatory state of mastitis in dairy cows via Gram staining of precipitates in milk. *Front. Vet. Sci.*10.3389/fvets.2024.1492564 (2025). 39872610 10.3389/fvets.2024.1492564 PMC11770005

[22] Moragas, A., Monfà, R., García-Sangenís, A. & Llor, C. Accuracy of leukocyte esterase and nitrite tests for diagnosing bacteriuria in older adults: A systematic review and meta-analysis. *Clin. Microbiol. Infect.* **32**(1), 19–29. 10.1016/j.cmi.2025.08.027 (2026). 40912457 10.1016/j.cmi.2025.08.027

[23] Jayalakshmi, J. & Jayaram, V. Evaluation of various screening tests to detect asymptomatic bacteriuria in pregnant women. *Indian J. Pathol. Microbiol.* **51** (3), 379. 10.4103/0377-4929.42516 (2008). 18723963 10.4103/0377-4929.42516

[24] Williams, G. J. et al. Absolute and relative accuracy of rapid urine tests for urinary tract infection in children: A meta-analysis. *Lancet Infect. Dis.* **10**(4), 240–250. 10.1016/S1473-3099(10)70031-1 (2010). 20334847 10.1016/S1473-3099(10)70031-1

[25] Patel, P. G., Raval, P., Shah, S. & Patel, K. Evaluating the predictive value of screening and confirmatory tests for asymptomatic bacteriuria in pregnant women attending a tertiary care hospital in Mehsana, North Gujarat. *Indian J. Microbiol. Res.* **12**(2), 193–198. 10.18231/j.ijmr.2025.027 (2025).

[26] Alonso-Tarrés, C. et al. Bacteriuria and phenotypic antimicrobial susceptibility testing in 45 min by point-of-care Sysmex PA-100 System: First clinical evaluation. *Eur. J. Clin. Microbiol. Infect. Dis.* **43**(8), 1533–1543. 10.1007/s10096-024-04862-3 (2024). 38825624 10.1007/s10096-024-04862-3 PMC11271345

[27] Collee, J., Fraser, A., Marmion, B. & Simmons, A. *Mackie & Mccartney Practical Medical Microbiology* 14th edn (Elsevier, 1996).

[28] Patel, P., Patel, M. & Desai, K. Prevalence of asymptomatic bacteriuria among pregnant women attending a tertiary care hospital in Western India. *Natl. J. Community Med.* **13**(10), 728–732. 10.55489/njcm.131020222444 (2022).

[29] Izewski, J. M., Bell, B. Z. & Haas, D. M. Antibiotics in Labor and Delivery. *Obstet. Gynecol. Clin. N. Am.* **50** (1), 137–150. 10.1016/j.ogc.2022.10.011 (2023). 10.1016/j.ogc.2022.10.011 36822699

[30] Rubenstein, J. N. & Schaeffer, A. J. Managing complicated urinary tract infections. *Infect. Dis. Clin. North Am.* **17**(2), 333–351. 10.1016/S0891-5520(03)00012-6 (2003). 12848473 10.1016/s0891-5520(03)00012-6

[31] Sordillo, E. M. & Polsky, B. Infections in Pregnancy. In *Principles of Gender-Specific Medicine* (pp. 531–562). Elsevier. (2010). 10.1016/B978-0-12-374271-1.00047-2

[32] Khapre, M., Sharma, D., Mehta, A. & Sinha, S. Prevalence of Asymptomatic Bacteriuria (ASB) in Pregnant Women in India: A Systematic Review and Meta-Analysis. *Indian J. Community Med.* **48** (6), 879–887. 10.4103/ijcm.ijcm_795_22 (2023). 38249695 10.4103/ijcm.ijcm_795_22 PMC10795867

[33] Sonkar, N., Banerjee, M., Gupta, S. & Ahmad, A. Asymptomatic bacteriuria among pregnant women attending tertiary care hospital in Lucknow, India. *Dubai Med. J.* **4**(1), 18–25. 10.1159/000513626 (2021).

[34] Abu, D., Abula, T., Zewdu, T., Berhanu, M. & Sahilu, T. Asymptomatic bacteriuria, antimicrobial susceptibility pattern and associated risk factors among pregnant women attending antenatal care in Assosa General Hospital, Western Ethiopia. *BMC Microbiol.* **21**(1), 348. 10.1186/s12866-021-02417-6 (2021). 34915840 10.1186/s12866-021-02417-6 PMC8675524

[35] Dereje, M., Woldeamanuel, Y., Asrat, D. & Ayenachew, F. Urinary tract infection among fistula patients admitted at Hamlin fistula hospital, Addis Ababa, Ethiopia. *BMC Infect. Dis.* **17** (1), 150. 10.1186/s12879-017-2265-4 (2017). 28209132 10.1186/s12879-017-2265-4 PMC5314475

[36] Mokube, M. N., Atashili, J., Halle-Ekane, G. E., Ikomey, G. M. & Ndumbe, P. M. Bacteriuria amongst pregnant women in the Buea Health District, Cameroon: Prevalence, predictors, antibiotic susceptibility patterns and diagnosis. *PLoS One* **8**(8), e71086. 10.1371/journal.pone.0071086 (2013). 23976983 10.1371/journal.pone.0071086 PMC3745459

[37] G, D., Sethuraman, D. & Revwathy, S. Study of association of urinary tract infection with preterm labour. *Indian J. Obstet. Gynecol. Res.* **7** (4), 567–572. 10.18231/j.ijogr.2020.121 (2020).

[38] Nteziyaremye, J. et al. Asymptomatic bacteriuria among pregnant women attending antenatal care at Mbale Hospital, Eastern Uganda. *PLoS One* **15**(3), e0230523. 10.1371/journal.pone.0230523 (2020). 32191758 10.1371/journal.pone.0230523 PMC7082119

[39] Antonellis, M., Adler, R., Kozlov, M., Lazar, J. & Weiss, J. P. Nocturia and SGLT2 inhibitors: A review of pathophysiology, clinical evidence, and therapeutic implications. *Continence* **16**, 102299. 10.1016/j.cont.2025.102299 (2025).

[40] Carpenter, R. E. Metabolic and microbial crossroads: Sodium-glucose cotransporter-2 inhibitors and urinary tract infections in (Asian) diabetes care. *Health Sci. Rev.* **15**, 100226. 10.1016/j.hsr.2025.100226 (2025).

[41] Raichoudhury, R. & Spinowitz, B. S. Treatment of anemia in difficult-to-manage patients with chronic kidney disease. *Kidney Int. Suppl.* **11**(1), 26–34. 10.1016/j.kisu.2020.12.006 (2021). 10.1016/j.kisu.2020.12.006 PMC7983023 33777493

[42] Sama, S. O. et al. Anaemia, iron deficiency and inflammation prevalence in children in the Mount Cameroon area and the contribution of inflammatory cytokines on haemoglobin and ferritin concentrations: A cross sectional study. *BMC Nutr.* **9**(1), 94. 10.1186/s40795-023-00748-3 (2023). 37507740 10.1186/s40795-023-00748-3 PMC10375674

[43] Choi, J. et al. Gut microbiome correlates of recurrent urinary tract infection: A longitudinal, multi-center study. *eClinicalMedicine* **71**, 102490. 10.1016/j.eclinm.2024.102490 (2024). 38813445 10.1016/j.eclinm.2024.102490 PMC11133793

[44] Jones-Freeman, B. et al. The microbiome and host mucosal interactions in urinary tract diseases. *Mucosal Immunol.* **14** (4), 779–792. 10.1038/s41385-020-00372-5 (2021). 33542492 10.1038/s41385-020-00372-5

[45] Solh, T., Thomas, R. & Roman, C. Current diagnosis and management of urinary tract infections. *Phys. Assist. Clin.* **2**(2), 191–205. 10.1016/j.cpha.2016.12.003 (2017).

[46] Laurenzana, L., Fitzgerald, C. & Bennis, S. Pelvic pain and pelvic floor disorders in women. *Phys. Med. Rehabil. Clin. North Am.* **36**(2), 329–342. 10.1016/j.pmr.2024.11.014 (2025). 10.1016/j.pmr.2024.11.014 40210365

[47] Dubbs, S. B. & Sommerkamp, S. K. Evaluation and Management of Urinary Tract Infection in the Emergency Department. *Emerg. Med Clin North Am.* **37** (4), 707–723. 10.1016/j.emc.2019.07.007 (2019). 31563203 10.1016/j.emc.2019.07.007

[48] Walsh, C. & Collyns, T. Pathophysiology of urinary tract infections. *Surgery (Oxford)* **41**(5), 272–277. 10.1016/j.mpsur.2023.02.014 (2023).

[49] Casiano González, A. et al. A novel chaperone-effector-immunity system identified in uropathogenic *Escherichia coli* UMN026. *PeerJ* **12**, e17336. 10.7717/peerj.17336 (2024). 38784397 10.7717/peerj.17336 PMC11114119

[50] Aravena-Ramírez, V. et al. Genomic scan of a healthcare-associated NDM-1-producing Citrobacter freundii ST18 isolated from a green sea turtle impacted by plastic pollution. *J. Global Antimicrob. Resist.* **36**, 389–392. 10.1016/j.jgar.2024.01.006 (2024). 10.1016/j.jgar.2024.01.006 38266960

[51] Chen, J.-W., Lau, Y. Y., Krishnan, T., Chan, K.-G. & Chang, C.-Y. Recent advances in molecular diagnosis of *Pseudomonasaeruginosa* infection by state-of-the-art genotyping techniques. *Front. Microbiol.*10.3389/fmicb.2018.01104 (2018). 29892277 10.3389/fmicb.2018.01104 PMC5985333

[52] Mahfouz, A. Y., Abed, N. N., Abd-EL-Aziz, A. S. & Fathy, R. M. Green synthesis of gamma rays-induced melanin-based bismuth oxide nanoparticles for evaluation of the antibacterial and anti-virulence activities against extra-intestinal pathogenic bacteria. *World J. Microbiol. Biotechnol.* **41**(9), 319. 10.1007/s11274-025-04533-1 (2025). 40856931 10.1007/s11274-025-04533-1 PMC12380928

[53] Cheng, B., Zaman, M. & Cox, W. Correlation of Pyuria and Bacteriuria in Acute Care. *Am. J. Med.* **135** (9), e353–e358. 10.1016/j.amjmed.2022.04.022 (2022). 35580716 10.1016/j.amjmed.2022.04.022

[54] Lee, A. L. H., Leung, E. C. M., Lee, M. K. P. & Lai, R. W. M. Diagnostic stewardship programme for urine culture: Impact on antimicrobial prescription in a multi-centre cohort. *J. Hosp. Infect.* **108**, 81–89. 10.1016/j.jhin.2020.10.027 (2021). 33181278 10.1016/j.jhin.2020.10.027

[55] Yang, S.-D., Yang, C.-C., Chen, Y.-S. & Chang, S.-J. A performance comparison of the fully automated urine particle analyzer UF-5000 with UF-1000i and Gram staining in predicting bacterial growth patterns in women with uncomplicated urinary tract infections. *BMC Urol.* **21**(1), 24. 10.1186/s12894-021-00791-x (2021). 33579236 10.1186/s12894-021-00791-x PMC7881468

[56] Hadwan, M. H. et al. An improved method for measuring catalase activity in biological samples. *Biol. Methods Protoc.*10.1093/biomethods/bpae015 (2024). 38524731 10.1093/biomethods/bpae015 PMC10957919

